# Factors Associated With Adoption of Health Information Technology: A Conceptual Model Based on a Systematic Review

**DOI:** 10.2196/medinform.3106

**Published:** 2014-05-23

**Authors:** Clemens Scott Kruse, Jonathan DeShazo, Forest Kim, Lawrence Fulton

**Affiliations:** ^1^College of Health ProfessionsSchool of Health AdministrationTexas State UniversitySan Marcos, TXUnited States; ^2^Department of Health AdministrationVirginia Commonwealth UniversityRichmond, VAUnited States; ^3^School of BusinessBaylor UniversityWaco, TXUnited States; ^4^College of BusinessDepartment of CIS and Quantitative MethodsTexas State UniversitySan Marcos, TXUnited States

**Keywords:** electronic health record (EHR), electronic medical record (EMR), health information technology (HIT), medical information systems, computerized provider order entry (CPOE), adoption

## Abstract

**Background:**

The Health Information Technology for Economic and Clinical Health Act (HITECH) allocated $19.2 billion to incentivize adoption of the electronic health record (EHR). Since 2009, Meaningful Use Criteria have dominated information technology (IT) strategy. Health care organizations have struggled to meet expectations and avoid penalties to reimbursements from the Center for Medicare and Medicaid Services (CMS). Organizational theories attempt to explain factors that influence organizational change, and many theories address changes in organizational strategy. However, due to the complexities of the health care industry, existing organizational theories fall short of demonstrating association with significant health care IT implementations. There is no organizational theory for health care that identifies, groups, and analyzes both internal and external factors of influence for large health care IT implementations like adoption of the EHR.

**Objective:**

The purpose of this systematic review is to identify a full-spectrum of both internal organizational and external environmental factors associated with the adoption of health information technology (HIT), specifically the EHR. The result is a conceptual model that is commensurate with the complexity of with the health care sector.

**Methods:**

We performed a systematic literature search in PubMed (restricted to English), EBSCO Host, and Google Scholar for both empirical studies and theory-based writing from 1993-2013 that demonstrated association between influential factors and three modes of HIT: EHR, electronic medical record (EMR), and computerized provider order entry (CPOE). We also looked at published books on organizational theories. We made notes and noted trends on adoption factors. These factors were grouped as adoption factors associated with various versions of EHR adoption.

**Results:**

The resulting conceptual model summarizes the diversity of independent variables (IVs) and dependent variables (DVs) used in articles, editorials, books, as well as quantitative and qualitative studies (n=83). As of 2009, only 16.30% (815/4999) of nonfederal, acute-care hospitals had adopted a fully interoperable EHR. From the 83 articles reviewed in this study, 16/83 (19%) identified internal organizational factors and 9/83 (11%) identified external environmental factors associated with adoption of the EHR, EMR, or CPOE. The conceptual model for EHR adoption associates each variable with the work that identified it.

**Conclusions:**

Commonalities exist in the literature for internal organizational and external environmental factors associated with the adoption of the EHR and/or CPOE. The conceptual model for EHR adoption associates internal and external factors, specific to the health care industry, associated with adoption of the EHR. It becomes apparent that these factors have some level of association, but the association is not consistently calculated individually or in combination. To better understand effective adoption strategies, empirical studies should be performed from this conceptual model to quantify the positive or negative effect of each factor.

## Introduction

### Background

The US Government passed the Health Information Technology for Economic and Clinical Health (HITECH) act [1] to incentivize adoption of the electronic health record (EHR) and to assuage the short run (SR) effects of cost to the health care organization in the adoption process. The three phases of Meaningful Use consume information technology (IT) strategies in the SR because of the HITECH act’s timeline for health care organizations to qualify for monetary incentives [2,3].

Adoption of the EHR is a significant goal. International vernacular for the EHR varies; for example, electronic patient record, computerized patient records, electronic medical records (EMRs), and digital medical record. The defining difference, as defined by the Institute of Medicine, the health arm of the US National Academy of Sciences, focuses on the longitudinal and interoperable nature of the electronic patient record [4]. Without these capabilities, the patient record is greatly limited in scope. The longitudinal and interoperable nuances of the EHR are not the only significant advantages; there are eventual cost savings as well.

Studies estimate that adoption of the EHR could eventually save more than $813 billion annually, prevent 200,000 adverse drug events, and enhance the doctor-patient relationship through increased communication [5]. Unfortunately, these benefits are realized in the long run (LR), while the investment to adopt the EHR is expended in the SR. A large deficit in the SR could inhibit a health care organization’s ability to compete or survive in heavily competitive environment.

The environment of health care is unique in a competitive environment. The health care organization develops an organizational strategy based on the local environment. To increase an organization’s ability to compete, its strategy might also include cost reduction, and EHR adoption runs counter to this goal in the SR. The health care environment faces many sources of influence, including a reluctance to accept technology.

There has been a tremendous amount of research dedicated to the study of acceptance of technology, specifically the Technology Acceptance Model (TAM) [6]. More recent work has suggested modifications to the TAM that explain a perception of usefulness and intentions from the aspect of social influence and the cognitive instrumental process [7,8]. Several organizational theories have been developed. These focus on the sources of influence and the reason for their existence.

### Organizational Theories

Organizational theories address influence, but none adequately addresses the complexity of the health care organization. Payers, providers, and patients all control resources that exert influence. The nature of the competitive environment will also exert influence on decisions. External influence from those who control resources can be explained through resource dependence theory [9,10]. Internal and external influences can be explained by the Diffusion of Innovation Theory through its introduction of compatibility, complexity, trialability, observability, and relative advantage [11-13].

According to resource dependence theory, health care organizations with the greatest level of dependence on other organizations that control the resources will feel the greatest level of environmental influence on its decisions [14]. The Resource Dependence Theory describes an external interdependence of organizations. *External Control of Organizations*, [14], which is an adaptation of Resource Dependence Theory, provides good insight for this study. The authors’ premise is that the external environment creates a social context and plays an important role in how organizational decisions are made. The lack of absolute independence requires some degree of interorganizational exchange of goods or services [14]. As organizations build and negotiate relationships with each other in the exchange of resources, positions of power are established. No one organization can provide all of its own resources, so each organization becomes dependent on the other organizations that control the resources.

Similar to Resource Dependence, the Diffusion of Innovation Theory describes a social system that influences through communication channels [11-13]. Diffusion of Innovation attempts to explain how “an *innovation*, is *communicated* through *channels over time* among members of a *social system*” [13]. This theory accounts for 49-97% of variance in the rate of adoption of innovation through five factors: compatibility, complexity, trialability, observability, and relative advantage*.* These factors are sorted into three categories of a predictive model for EHR adoption: innovation determinants, organizational determinants, and environmental determinants [8]. The next several paragraphs exercise the five factors to this study.

The concept of compatibility [13] goes beyond answering the question, “is a product/service right for a market?” It also asks, “is the market ready for the product/service?” For instance, the Chevy Nova failed in Spanish-speaking markets because in Spanish the word “Nova” means “does not go.” Promotion of conservation techniques to farmers in the United States initially failed because farmers associated conservation with lower crop yield. Boiling water to sanitize it makes perfect sense to a market that is familiar with germ theory, but primitive tribes in Peru only heated water for sicker, weaker members; as a result, the concept failed when initially introduced and dysentery continued to flourish. In relation to this study, the concept of compatibility might ask, “is the market ready for the EHR?”

The concept of complexity [13] is appropriate to this study because innovation can be a double-edged sword. On one hand, it is new and may offer some improvement to a product or service. However, it might also be perceived as too complex; and perception can be a powerful force. If the Baby Boomer generation perceives computers to be too complex, and this perception causes computer anxiety, its users may reject its adoption and use [15]. The older physicians in a hospital have greater seniority, and are therefore, more influential in the hospital’s decision to adopt the EHR. Would this same generation of providers influence the health care organization considering EHR adoption?

The concept of trialability [13] applies more to the early-adopter group than the other groups: innovators, early-majority, late-majority, and laggards. In the early phase of promotion for a new product or service, the vendor might lower the risk of adoption by offering free trials or samples to potential users. Once the user is confident of the new item’s efficacy, then he/she is more likely to pay full price for its use. When a new producer of an EHR enters the marketplace, it must incentivize the use of its product because it is not known in the industry. The user accepts a risk by trying the new EHR, but the risk is overcome by the incentive. Once the new EHR gains momentum in the industry, adoption enters the early-majority phase. The new EHR has already gained momentum in the industry, and the producer does not need to incentivize its use.

The concept of observability [13] is also highly applicable to this study. Decision makers in a hospital that has not yet adopted an EHR will observe the experiences of other hospitals that have adopted it. Vendors will promote or advertise specifically to the nonadopters and help them observe how the EHR can benefit its organization. External players in the health care organization’s competitive environment will provide some level of observability.

Relative advantage is a multifaceted concept for this study. In health care, the most important factor is provision of health, as well as the treatment and prevention of disease. If adoption of the EHR speaks directly to the health care organization’s primary purpose, then it might provide relative advantage over competitors that have not adopted it. Another concept is that of social prestige [13]. Unless a health care organization can serve as an example to other health care organizations (observability), there may not be a sufficient level of relative advantage to be considered.

### Strategy and Decision Making

Strategy can be a multifaceted concept, and organizations around the world hire strategy experts to help identify and focus on a market forces. An operational definition of strategy is borrowed from education [16] and is adapted to health care: strategy is defined as instruments by which *health care organizations* manage their organizational processes and deal with their environments in order to select a portfolio of activities and find appropriate position in the *health care industry* (italics indicate a change in wording from the authors’ definition). It follows that adoption of an EHR would alter how a health care organization manages its organizational processes, so this definition of strategy is a good fit for the health care industry. However, two significant considerations in the health care environment are the level of local competiveness, and how health care organizations compete [17].

Studies have shown that decision making in the health care industry is often based on how the organization competes, whether in a single-market or multimarket environment [18]. In either environment, decision-making varies on competition, and the health care industry competes in clusters [18]. The way health care organizations compete will also affect its organizational structure. A four-cluster solution was identified as a reliable, internally valid, and stable model for health networks and a five-cluster solution for health systems [19]. Differentiation and centralization are particularly important in distinguishing unique clusters of organizations. High differentiation typically occurs with low centralization, which suggests that a broader scope of activity is more difficult to centrally coordinate. Integration is also important, but the authors find that health networks and systems typically engage in both ownership-based and contractual-based integration or they are not integrated at all.

Ash and Bates [20] studied the EHR adoption rates and the factors and forces affecting system adoption through surveys (85/650, 13.1%). Only 106 of the 650 (16.3%) of hospitals surveyed had adopted some form of EHR, 63/106 (59.4%) had implemented a full Computerized Provider Order Entry (CPOE) solution, and the other 43/106 (40.6%) implemented a partial CPOE solution. A full one-third of adopters were either Veterans Affairs or military hospitals. Additionally, 481/650 (73.8%) of those who planned to implement a full solution intended to do so within 5 years. Ash and Bates [20] also found that the size of hospital is positively-associated with component adoption; specifically CPOE adoption. The authors inferred from their results that the primary reasons to adopt the EHR is to gain the quality-of-care advantages of CPOE. This inference reinforced our inclusion of CPOE as a dependent variable.

Factors that influence health information system (HIS) adoption in US hospitals have been studied by others as well (n=1441) [21]. Results showed that HIS adoption is influenced by the hospital market, organizational, and financial factors. Larger, system-affiliated, and for-profit hospitals with more preferred provider organization contracts are more likely to adopt managerial information systems than other hospitals. Operating revenue is positively associated with HIS adoption. The study also identified hostility as an aspect of environmental uncertainty, and that organizations often turn to technological adoption to regain competitive advantage.

A knowledge-based taxonomy of critical factors for adopting an EHR was developed from a systematic literature review [22]. The researchers selected 68 of 3400 (2.00%) articles to identify six factors of adoption, listed in order of importance: user attitude toward information systems, workflow impact, interoperability, technical support, communication among users, and expert support.

Alternative measures of EHR adoption among hospitals have been studied [23]. Authors analyzed a 2009 information technology supplement survey distributed by the American Hospital Association (AHA). The survey focused on 24 EHR functionalities in various areas: electronic clinical documentation, results viewing, CPOE, and clinical decision support. They found that 142 of 3937 (3.60%) acute-care hospitals in the United States of responding hospitals have implemented all 24 functions, 386/3937 (9.80%) of hospitals have implemented at least 20 functions, and 1437/3937 (36.50%) have implemented at least one-half of the functions. The researchers added that EHR adoption is a complex process.

Others have studied the relationship between hospital financial position and the adoption of the EHR [24]. Through a cross-sectional study of secondary data from several sources, including the AHA (2442/5752, 42.51% acute-care hospitals in the United States), researchers identified five independent and one dependent variable. Of the five independent variables (IVs), only liquidity was positively-associated with EHR. Asset turnover was negatively-associated with EHR adoption. Bed size, a control variable, was positively-associated with EHR adoption. The authors concluded that hospitals adopt EHRs as a strategic move to better align themselves with their environment.

Because commonly used elements of organizational strategy are difficult to change, several of the variables were categorized as internal organizational factors. Research has assessed variables of hospital influence in five categories: (1) capacity as measured by number of beds in groupings by intervals of 100, (2) management, or ownership, (3) organizational focus, or teaching status, (4) competitive location and alternatives, and (5) state regulatory pressures [25].

Although resources have been consumed to study factors associated with adoption of HIT, there is a gap in the literature that provides a conceptual model to guide the design of empirical models. It may seem backward to design a conceptual model after so many studies have already been conducted, but the gap remains. The aim of this study was to develop a conceptual model from a systematic literature review that associates both internal and external factors associated with adoption of the EHR. The intent of the conceptual model is to enable future empirical models.

## Methods

### Literature Review Process

Search terms were selected based on the experience of the authors in the field of health care administration. The time frame of 1993-2013 was selected as convenience. It was assumed that 2 decades would be sufficient to capture trends.

**Figure 1:**
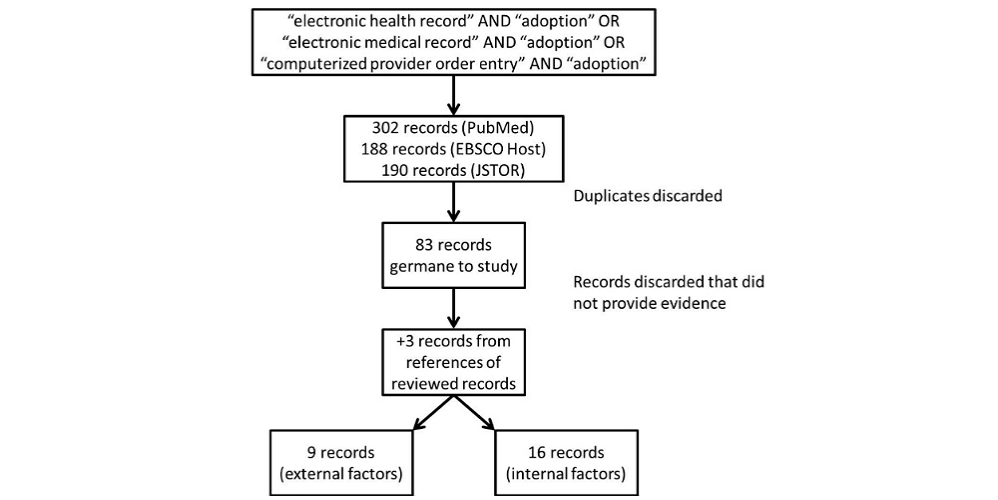
Literature review process.

Figure 1 illustrates the literature review process that identified 83 sources consisting of empirical studies, articles, editorials, commentaries, opinion papers, organizational theories, and text books. The intent of no limits to the type of papers was to mitigate the risk of missing something significant from a study that was not catalogued properly within a key word catalogue like the Dublin Core.

The 83 records were reviewed for content and evidence. After discarding 58 articles for lack of evidence, three additional references were added because they were key concepts upon which other studies were based. Of the remaining 25 articles, a list of factors was identified as IVs. Some factors were grouped under a similar category for the purposes of simplification of the conceptual model. The dependent variable (DV) started as adoption of the EHR, but the studies from those chosen were not as specific. From personal experience, many studies seem to discuss the EHR, but call it something else: most commonly the EMR. That is why EMR was included in the search. Because so few ERHs exist without some form of CPOE, the latter term was included in the search criteria.

Our study combines the influences highlighted by previous work and examines determinants of EHR adoption. Examining EHR adoption at the health care organization level will demonstrate validity between this study and others that have used the hospital as the unit of analysis.

### EHR Adoption and Internal Organizational Influence

Several influences in the environment exert pressure on the health care organization to adopt EHR. Influences range from incentives from the federal government to the nature of local competitive community. US federal incentives provide a heavy influence for EHR implementation, under specific conditions, and imposes penalties for a lack of EHR implementation.

The internal politics of one organization serve as one source of influence. A hospital is part of a community, which serves as an external influence. Further, if a hospital is also part of a larger multihospital system (MHS), then the politics of the broad MHS will also exert influence on local decisions.

### EHR Adoption and External Environmental Influence

The patient is external to the organization, and for our study, the patient primarily serves as an external influence. Although some employees of the health care organization might also be patients, and this relationship could create a small internal influence, this study considers those few stake holders in the internal organizational factor of users. The providers are internal to the organization, and for our study, providers serve as an internal organizational influence. The payer is a significant influence [14], and the Center for Medicare and Medicaid Services (CMS) serves as a good example of this significant influence [26]. The HITECH act provides monetary incentives for EHR adoption. Those who do not implement all aspects specified in the stages of adoption are not eligible for the incentives. In this way, the CMS disincentivizes those organizations that do not adopt the EHR. If payments from the CMS were of little consequence to the health care organization’s revenue, then the health care organization might decide differently about EHR adoption. A competing health care organization is an external market force in the environment. Third-party payers might compare health care organizations based on maturity of automation because mature clinical components like CPOE will result in more accurate billing. Such forces incentivize a health care organization to adopt the EHR.

### Overview of the Conceptual Model

The premise for an EHR adoption conceptual model is that that environmental influences affect organizational strategy of the health care organizations that adopt the EHR [13,14,20,22,24,25]. Diffusion of Innovation theory provides three categories of a predictive model for EHR adoption: innovation determinants, organizational determinants, and environmental determinants [13]. Resource Dependence Theory provides a category of a predictive model for EHR adoption, the competitive environment. In construction of the EHR adoption conceptual model, several constructs emerged [14].

Elements of organizational strategy are not variables that can be easily changed [19]; therefore, elements typically ascribed to strategy, such as size, ownership, and fiscal stability, will be absorbed into the IVs of influence. This research proposes a model, whereby environmental factors are associated with an organization’s decision to adopt the EHR.

Resource Dependence Theory explains environmental influences and the external interdependence of organizations [14]. The authors’ premise is that the external environment creates a social context and plays an important role in how organizational decisions are made. The interdependence of organizations widens the field of stakeholders, and this relationship effect should be defined.

Disparate stakeholders have different interests with reference to different components of the EHR. These interests may be different in the SR interests versus the LR interests. SR interests are those that are immediate, such as current year expenditures. LR interests are further out when all inputs are variable. The SR interests of cost can often compete with the LR potential of cost savings and greater safety. Both the SR and LR interests are affected by the external environment [17].

In a highly competitive environment, SR cost implications could often win over any long-term savings. The number of patients in a market is fixed in the SR, and a highly competitive market will affect each competitor’s share of that market. The SR costs of EHR implementation might be insurmountable by an organization in this market because it could not afford to lose ground without significant capital reserves or the ability to borrow cheaply [17]. However, in a less competitive market, the LR interests of potential cost savings have a better chance of influencing the decision to implement an EHR because the costs incurred in the SR are justified by the long-term benefits [17].

External stakeholders that control resources important to the health care organization can exert significant influence. For instance, a health care organization that receives a significant amount of revenue from the CMS will be influenced more by incentives provided by the CMS than an organization that receives a significant cash flow from private third parties. The relative influence of various external stakeholders may be captured by an analysis of the structure of the market in which a health care organization operates.

Stakeholders have varying interests with regard to the capabilities and effects of EHR components depending upon their relationship with the health care organization. Private payers have both SR and LR interests in the EHR. In the SR, their focus is on minimizing expenditures. Because the health care organization would pass on the implementation costs through higher contract costs, payers would not be equal in the SR. In addition, the disruption of EHR implementation could potentially affect care processes and therefore increase claims. Payers would be interested in the LR benefits of the EHR: potential cost savings, better disease management, and increased safety. However, the SR interests of the private payers might overshadow the LR benefits of the EHR. Public payers enable care of the indigent and elderly. As part of the United States Department of Health and Human Services (HHS), the CMS is highly interested in disease management, public health, safety, and research, and it may value these LR capabilities of the EHR more than the SR costs. The CMS, as part of HHS, would also favor the EHR because it supports the Presidential directive to promote the establishment of the Nationwide Health Information Network that links electronic patient records through health information exchanges.

Providers and patients value face time with each other. During EHR implementation, providers might spend less time in communication with patients. Providers must adapt their processes and clinic-to-administrative schedules. Any disruption or action that is perceived as deleterious to this relationship could result in a negative reaction to EHR implementation. As a result, physicians might oppose EHR adoption, or they might simply support the EHR solution with the shortest implementation time or least administrative burden. Patients might not like the reduced face time with the provider, but they might be attracted to EHR components such as e-prescribing, e-results, personal health records, and email access to the provider. These desirable features are available to the patient when the health care organization chooses to adopt various portions of the CPOE component to the EHR.

## Results

### Chosen Articles

The articles chosen for final inclusion were read once more to make a list of variables. The variables from the studies were listed as internal and external. There were significant commonalities in the variables used, so they were combined in the model.

### EHR Adoption and Internal Organizational Influence

As depicted in Figure 1, 16 references identified internal factors [7,8,12,14,15,19,21-24,27-33]. Six identified size of the health care organization, and six identified strategic alliances. Five identified ownership and five identified complexity of care. Four identified capital expenditures. Three identified users, and three identified teaching status. Two identified user attitude toward HIS, and two identified communication among users. Workflow impact, interoperability, technical support, expert support, physician arrangements, unity of effort, and user computer anxiety were all identified by one study, independently.

The dependent variable was not consistent: seven references used EHR adoption [23-25,28,30,31,33], two used “electronic capture of clinical data,” [23,25] one used a generic DV of technology adoption [15], and six used CPOE [20,23,25,28,29,31].

### EHR Adoption and External Environmental Influence

As depicted in Figure 1, nine studies identified external environmental factors [12,14,15,19,22,24,28,30,31]. Five studies identified buyers, four studies identified patients, three studies identified competitiveness, two studies identified location, and one identified interdependence factors external to the organization that are associated with adoption of the EHR.

### Overview of the Conceptual Model

**Figure 2:**
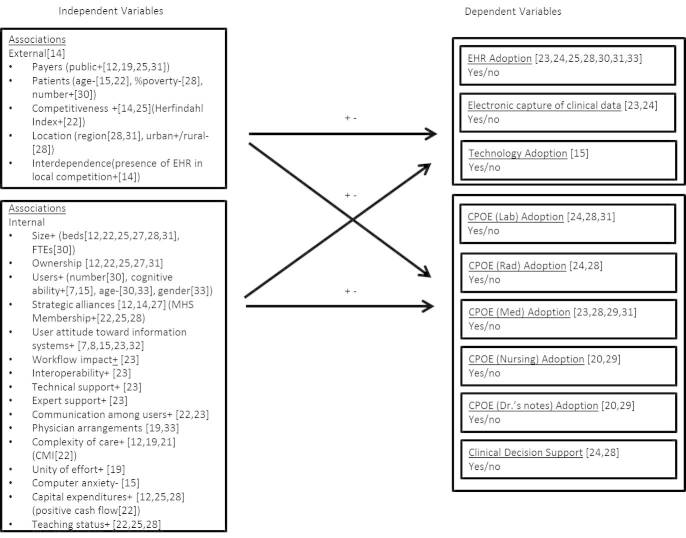
Conceptual model of factors associated with adoption of the EHR.

As previously stated, there was overlap between the sources/theories. There were four internal forces and seven external forces identified through multiple works by three authors [11-14,22]. However, it was unclear in existing literature the degree to which these forces can influence a health care organization’s decision to adopt the EHR. A complex conceptual model should provide insight into the strength and direction of the influence on the complex health care organization. The resulting conceptual model, depicted in Figure 2, posits a complex relationship between environmental influences, organizational strategy, and EHR adoption.

This framework captures both internal and external factors that influence the adoption of the EHR. The positive (+) and negative (−) signs in the model describe the relationship identified by the associated authors. For instance, Gin et al [24] identified a positive relationship between the external environmental factors of public payer (IV) and competitiveness (IV) and an association with the adoption of an EHR (DV). That is to say, the greater the percentages of an organization’s reimbursements that come from a public source like CMS, the stronger the association of the organization’s adoption of the EHR. Likewise, the greater the Herfindahl Index of the local competitive environment, the stronger the association of the organization’s adoption of the EHR. Age is another interesting factor because of its negative relationship with adoption. The older the patient population (external environmental IV) [15] and provider population (internal organizational IV) [30], the lower association with the adoption of the EHR (DV). The + and − signs above the arrows between the IVs and DVs indicates the variety of positive and negative associations with the adoption of an EHR.

## Discussion

### Principal Findings

The main findings of this study were that nine studies identified external factors and 16 studies identified internal factors associated with the adoption of EHR. These factors were depicted in a conceptual model to describe relationships to EHR adoption.

The conceptual model for EHR adoption illustrates a framework within which both administrators and policy makers can work to understand the levers that exert significant influence in the adoption of EHR. The extensive literature review conducted by this study builds a unique model from which empirical studies can be designed.

Identifying relationships between the adoption factors and adoption of the EHR becomes significant because it identifies levers that will produce a desired action. For instance, if a hospital has a majority of senior providers, perhaps from the Baby Boom generation, the administrators become aware of the additional effort that needs to go into user acceptance. A hospital that has a majority of new providers will not need to expend the resources on user acceptance, because studies already show a penchant for technology in younger generations. Similar inferences on other factors of adoption could be made, and some would require additional study.

For instance, the literature on workflow impact is split. There seems to be evidence that the presence of the EHR both enhances and complicates the providers’ workflow. This observation clearly begs additional questions. Were subjects for the data in different phases of adoption of the EHR? Was the hospital that responded negatively in the middle of an EHR implementation? Logically, a large implementation of any technology will become disruptive to the organization. Several studies could emerge from this relationship alone.

Empirical models could easily be designed to further investigate specific relationships between the IVs and DVs. The set of studies on CPOE was interesting. Although there were some overlaps with adoption of the EHR, there were also studies that only looked at CPOE. There does not seem to be an abundance of evidence in the literature about CPOE, and yet the AHA regularly collects data on six different versions of CPOE: laboratory, radiology, pharmacy, nursing, physician notes, and consults. There was no data to be found about the use or efficacy on CPOE consults. It might be interesting to determine the reason for this paucity of data.

### Limitations

The EHR adoption conceptual model associates internal and external factors with the adoption of the EHR, but it is primarily based on an extensive literature review. So far, it is not empirically tested. However, data are available to test the theory. Because the findings of our study are descriptive in nature, we do not opine on appropriate medical use of the information.

Caution should be identified with the interaction of variables. Some variables will most likely confound or mask the effects of others. For instance, is there a direct relationship between the number of beds of a hospital and the number of full-time equivalents? There are staffing models that would most likely answer that question. If there is a similar relationship, then one of these variables should be eliminated in favor of the stronger one. Otherwise, the effects of the weaker variable will be masked by the other. A false conclusion could easily be identified concerning the masked variable.

A majority of references for this study were from the United States, with one exception from Hong Kong. The internal validity of this study is strongest within the US health care sector. The conceptual model might be limited outside the United States because of the nature of competition between hospitals. The analysis of 83 articles identified studies that used similar methods: survey or secondary data analysis. Many authors analyzed data from the AHA, a well-established data set in the United States. These data are self-reported, which comes with limited bias.

### Conclusions

This study also identified overlap between studies in terms of variables. One interpretation of this overlap could be that the variables and associated studies are highly reliable. The key word/phrase searches described in the Methods section identifies the databases queried and results given. Other researchers should be able to duplicate or update this conceptual model going forward.

## Abbreviations

AHA: American Hospital Association
ARRA: American Recovery and Reinvestment Act
CMS: Center for Medicare and Medicaid Services
CPOE: computerized provider order entry
DV: dependent variables
EHR: electronic health record
EMR: electronic medical record
HHS: the Department of Health and Human Services
HIMSS: Health Information Management Systems Society
HIS: health information system
HIT: health information technology
HITECH: Health Information Technology for Economic and Clinical Health
IT: information technology
IV: independent variables
LR: long run
MHS: multihospital system
SR: short run
TAM: Technology Acceptance Model
